# Optimization of Extraction Technology of Majun Mupakhi Ela and its Effect on Hydrocortisone-induced Kidney Yang Deficiency in Mice

**DOI:** 10.1038/s41598-019-41006-6

**Published:** 2019-03-15

**Authors:** Ayinuer Reheman, Ze-yu Gao, Xirali Tursun, Xiao-Ping Pu, Tao Wu, Fei He, Xin Zhao, Haji Akber Aisa

**Affiliations:** 1 Key Laboratory of Plant Resources and Chemistry in Arid Regions, Xinjiang Technical Institute of Physics and Chemistry, Chinese Academy of Sciences, 830011 Urumqi, Xinjiang China; 2 College of Traditional Uyghur Medicine, Xinjiang Medical University, 830011 Urumqi, Xinjiang China; 3 Department of Molecular and Cellular Pharmacology, School of Pharmaceutical Sciences, Peking University, Xue yuan Road 38, Beijing, P. R. China; 4 University of Chinese Academy of Sciences, 100039 Beijing, China

## Abstract

We used Box-Behnken design-based (BBD) response surface methodology (RSM) in this research to optimize the extraction process of Traditional medicine Majun Mupakhi Ela (MME) and evaluate its effect on hydrocortisone-induced kidney yang deficiency. Three independent parameters were applied to evaluate the maximum phosphodiesterase type 5 (PDE5) inhibition activity of MME extracts *in vitro*. The optimal processing conditions (extraction time 2 h, solid-liquid ratio 1:16, extraction once) gave a maximum PDE5 inhibition rate of 84.10%, flavonoid content of 0.49 mg/ml, icariin content of 0.028 mg/ml and targeted extraction yield of 26.50%. In animal experiments, MME extracts significantly increased the adrenal mass index, semen weight index, preputial gland weight index, and penis weight index in mice; in the middle and high dose group, the level of serum testosterone increased by 7664.29% and 14207.14% respectively, compared with the model group, and the level of PDE5 decreased by 67.22% and 74.69% respectively compared with the control group. These results indicate that MME has a significant positive effect on the hypothalamus-pituitary-gonadal axis, improve mating ability and not only has inhibits PDE5 activity but also significantly inhibits the expression of PDE5 in penile tissues, potential to become erectile dysfunction (ED) therapies for the clinical management of patients with kidney yang deficiency.

## Introduction

The definition of erectile dysfunction (ED) is the persistent inability to obtain and maintain an erection sufficient to realize naturally satisfying intercourse. Male impotence, also called ED, is highly prevalent and affects nearly half of men aged 40 to 70, corresponding to around 150 million men worldwide^1^. Clearly, ED is considered to be a major health issue for the growing healthy aging population.

The cellular enzyme PDE-5 can be inhibited to reduce the breakdown of cyclic guanylate monophosphate, which promotes penile erection during sexual intercourse and stimulation of vascular relaxation in the corpora cavernosa^2^.

PDE5 inhibitors have been used to treat ED since 1998, when sildenafil was introduce^3,4^. People are dependent on synthetic products, such as tadalafil (sold most commonly as Cialis) and sildenafil (sold most commonly as Viagra), for ED treatment^5–7^. However, these substances can have negative side effects, such as blurred vision, muscle pain, and headache, and may have risky interactions with many other medications^8^. The search for natural substances that have the ability to promote the sexual experience with no negative side effects has been increased by the capacity of these synthetic products for treating ED^9^.

Accompanied by diverse pathophysiological changes within organisms^10–13^, Kidney Yang Deficiency Syndrome (KDS-Yang) was documented within Huang di Nei jing for the first time. KDS-Yang is part of a diagnostic pattern within Chinese medicine similar and has similar clinical characteristics to glucocorticoid withdrawal syndrome^14,15^. It has been shown by modern research that functional disorders and damage mediated through the hypothalamic-pituitary-target gland axis, containing the gonads, thyroid, and adrenal glands, are the major pathological mechanism of KDS-Yang^15^.

Traditional medicine MME is administered in the form of a Majunat (cream) composed of *Epimedium brevicornu* Maxim, *Anacyclus pyrethrum* (L.) DC, *Lycium barbarum* L, *Cuscuta auctralis* R.Br, *Rhodiola crenulata* (Hook.f.et Thoms) H.Ohba, *Cinnamomum cassia Presl*, *Orchis morio*.L, *Polygonatum odoratum (Mill) Druce*, and *Crocus sativus* L. MME has been used as an aphrodisiac and to treat both impotence and erectile dysfunction in clinical settings^16^. Because its dosage form is Majunat (a cream), there are some short-comings of using MME, such as inconvenience for transportation, poor taste, requirement of a large amount, and difficult to control content, and in addition, extensive pharmacological investigations of MME have not been conducted.

Every medicinal plant study uses extraction as the first procedure, which plays an important role in the ultimate results^17^. Most extraction techniques investigate extraction with various solvents in combination or alone as well a heat as the base. Carefully selecting the extraction parameters can result in an increase of the yield of the target molecule with minimal cost.

Industry regards an increase of final product quality and reduction of production cost as important. To achieve this goal, diverse optimization approaches can be successfully adopted. Response surface methodology (RSM) depends on statistical and mathematical methods to define process parameters’ optimum values by realizing desirable response(s). Optimization of extraction procedures of bioactive compounds widely uses RSM^18–21^.

Therefore, in this study, we optimized the extraction process to increase the stability and convenient use of MME as well as to further demonstrate its aphrodisiac and anti-erectile dysfunction (PDE-5 inhibition activity) capacities through animal experiments.

This study aimed at establishing optimized extraction conditions to develop extracts with a maximum PDE5 inhibitory activity and evaluate their effect on hydrocortisone induced kidney yang deficiency.

## Results and Discussion

### Correlation analysis

The design of the experiments was in accordance with RSM design. Table 1 presents the results. The effect on response according to quadratic, interaction and linear coefficients was tested by analysing variance for significance. Table 2 presents the regression coefficients of the linear, intercept, and cross product, as well as the quadratic terms. Variance analysis was also used to analyse the suitability of this model. Table 3 shows the calculated statistical parameters.

**Table 1:** Experimental matrix and values of the observed responses.

Run	X_1_ (h)	X_2_ (multiple)	X_3_ (times)	Flavonoid content (mg/ml)	Icariin content (mg/ml)	Extraction yield (%)	PDE5 inhibition rate (%)
1	1 (−1)	16 (1)	2 (0)	0.51	0.0155	26.80	70.4
2	1 (−1)	14 (0)	1 (−1)	0.38	0.0265	21.31	49.5
3	1 (−1)	12 (−1)	2 (0)	0.52	0.0209	26.71	82.1
4	1 (−1)	14 (0)	3 (1)	0.33	0.0170	29.22	77.4
5	2 (1)	12 (−1)	2 (0)	0.33	0.0208	28.19	72.5
6	1.5 (0)	14 (0)	2 (0)	0.55	0.0095	27.92	36.6
7	2 (1)	16 (1)	2 (0)	0.61	0.0181	31.09	64.0
8	2 (1)	14 (0)	3 (1)	0.67	0.0199	32.02	74.0
9	1.5 (0)	12 (−)	3 (1)	0.21	0.0085	28.97	64.7
10	1.5 (0)	14 (0)	2 (0)	0.53	0.0059	29.88	36.5
11	2 (1)	14 (0)	1 (−1)	0.49	0.0258	23.25	73.6
12	1.5 (0)	12 (−1)	1 (−1)	0.34	0.0168	21.35	61.7
13	1.5 (0)	16 (1)	1 (−1)	0.31	0.0197	23.14	63.3
14	1.5 (0)	14 (0)	2 (0)	0.54	0.0094	27.78	36.6
15	1.5 (0)	14 (0)	2 (0)	0.54	0.0096	28.88	36.4
16	1.5 (0)	14 (0)	2 (0)	0.52	0.0096	29.18	36.2
17	1.5 (0)	16 (1)	3 (1)	0.57	0.0014	32.42	81.0

X_1_ (h): extraction time; X_2_ (multiple): solid-liquid ratio; X_3_ (h): number of extractions.

**Table 2:** Regression coefficients of the predicted second-order model for the response variables.

Regression coefficient	Flavonoid content	Icariin content	Extraction yield	PDE5 inhibition rate
*β0*	0.54	8.800E-003	28.73	36.46
Linear				
*β1*	0.045	5.875E-004	1.31	0.59
*β2*	0.075	−1.537E-003	1.03	−0.29
*β3*	0.032	5.250E-003	4.20	6.13
Cross product				
*β12*	0.073	6.750E-004	0.70	0.80
*β13*	0.058	9.000E-004	0.22	−6.88
*β23*	0.098	−2.500E-003	0.42	3.68
Quadratic				
*β11*	0.033	0.010	−0.28	18.37
*β22*	−0.077	−3.375E-004	−0.26	17.42
*β33*	−0.10	3.3138E-003	−2.00	13.80
R^2a^	0.8525	0.9653	0.9794	0.9257
CV^b^	15.63	13.37	2.70	11.95

^a^Coefficient of multiple-determination. ^b^Coefficient of variance (%).

**Table 3:** Analysis of variance (ANOVA) for the fitted quadratic polynomial model.

Source	*SS*	*Df*	*MS*	F value	p value
**Total flavonoid content**					
Model	0.22	9	0.024	4.50	0.0301
Residual	0.037	7	5.346E003		
Lack of fit	0.037	3	0.012	94.62	0.0004
Pure error	5.2004E-004	4	1.300E-004		
Total	0.25	16			
Model equation	Y = 0.54 + 0.045X_1_ + 0.075X_2_ + 0.032X_3_ + 0.073X_1_X_2_ + 0.058X_1_X_3_ + 0.098X_2_X_3_ + 0.033${{\rm{X}}}_{1}^{2}$ − 0.077${{\rm{X}}}_{2}^{2}$ − 0.10${{\rm{X}}}_{3}^{2}$				
**Icariin content**					
Model	7.824E-004	9	8.693E-005	21.63	0.0003
Residual	2.813E-005	7	4.019E-006		
Lack of fit	1.759E-005	3	5.864E-006	2.23	0.22761
Pure error	1.054E-005	4	2.635E-006		
Total	8.105E-004	16			
Model equation	Y = 8.800E − 003 + 5.875E − 004X_1_ − 1.537E − 003X_2_ + 5.250E-003X_3_ + 6.750E-004X_1_X_2_ + 9.000E-004X_1_X_3_ − 003 − 2.500E-003X_2_X_3_ + 0.010${{\rm{X}}}_{1}^{2}$ − 3.375E − 004${{\rm{X}}}_{2}^{2}$ + 3.3138E − 003${{\rm{X}}}_{3}^{2}$				
**Extraction yield**					
Model	184.19	9	20.47	36.95	< 0.0001
Residual	3.88	7	0.55		
Lack of fit	0.77	3	0.26	0.33	0.8046
Pure error	3.11	4	0.78		
Total	188.07	16			
Model equation	Y = 28.73 + 1.31X_1_ + 1.03X_2_ + 4.20X_3_ + 0.70X_1_X_2_ + 0.22X_1_X_3_ + 0.42X_2_X_3_ − 0.28${{\rm{X}}}_{1}^{2}$ − 0.26${{\rm{X}}}_{2}^{2}$ − 2.00${{\rm{X}}}_{3}^{2}$				
**PDE5 inhibition rate**					
Model	4452.56	9	494.73	9.70	0.0034
Residual	357.14	7	51.02		
Lack of fit	357.03	3	119.01	4250.39	0.0001
Pure error	0.11	4	0.028		
Total	4809.71	16			
Model equation	Y = 36.46 + 0.59X_1_ − 0.29X_2_ + 6.13X_3_ + 0.80X_1_X_2_ − 6.88X_1_X_3_ + 3.68X_2_X_3_ + 18.37${{\rm{X}}}_{1}^{2}$ + 17.42${{\rm{X}}}_{1}^{2}$ + 13.80${{\rm{X}}}_{3}^{2}$				

SS: Sum of squares; Df: Degrees of freedom; MS: Mean of square.

### RSM analysis and HPLC analysis

**Figure 1:**
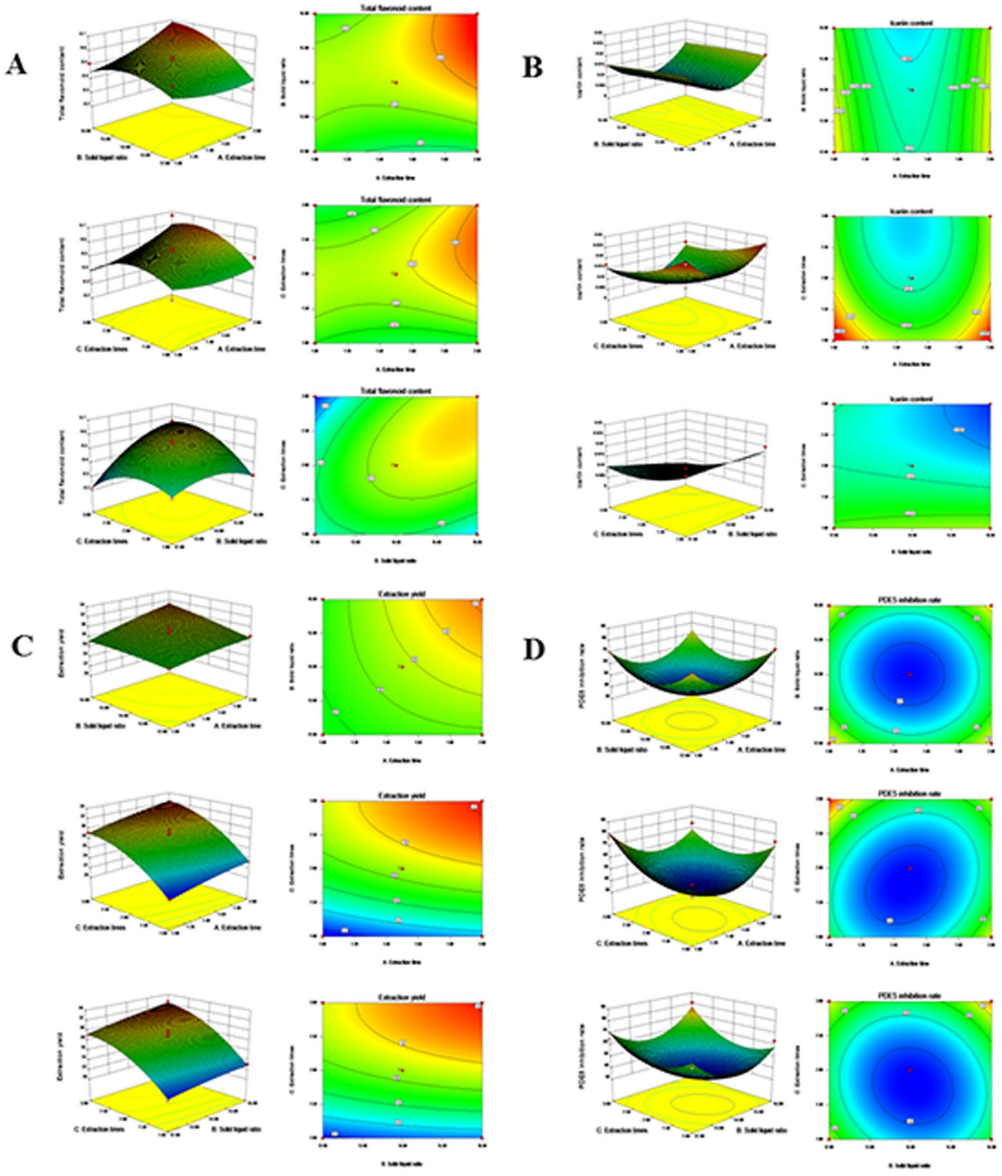
Response surface plots showing the effects of the investigated parameters on the flavonoid content, icariin content, extraction yield, and PDE5 inhibition rate (**A**) total flavonoid content; (**B**) icariin content; (**C**) extraction yield; (**D**) PDE5 inhibition rate.

**Figure 2:**
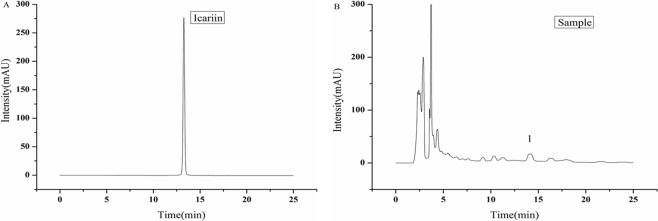
HPLC chromatogram of icariin in the standard (**A**) and sample (**B**). (The retention times are 13.256 and 13.469).

According to the quadratic polynomial equation, the response curves and contour plots of the interaction items of each index and response value were drawn to determine the influence of each factor on the total flavonoid content, icariin content, extraction yield and inhibition rate of PDE5; the results are shown in Fig. 1, HPLC chromatogram of icariin in the standard and sample shown in Fig. 2

### Validation of the MME extraction process

The major purpose of this study was to identify the best conditions regarding the extraction numbers, solid-liquid ratio and extraction time within the experimental scope, as shown in Table 2. The use of second-order polynomial models for every response was to determine the optimal extraction conditions. The icariin content, extraction yield and PDE5 inhibition rate were optimized by applying the RSM total flavonoid content. The optimal system was identified for every response. The predicted results and experimental values of the response variables under a given condition are listed in Table 4.

**Table 4:** Predicted and experimental values of the response variables under a given condition within the range of the optimum extraction conditions.

Response variables	Predicted value	Experimental values
Total flavonoid content (mg/ml)	0.47	0.49
Icariin content (mg/ml)	0.026	0.028
Extraction yield (%)	26.00	26.50
PDE5 (%)	81.07	84.10

Given conditions: 2 h extraction time, 1:16 in solid-liquid ratio, 1.19 times extraction number.

### Effects of the extraction parameters on the total flavonoid content

The data was fitted by Design-Expert 8.06. The following equation was used to describe the effects due to extraction time, solid-liquid ratio and extraction numbers on the overall flavonoid content of MME:

$$\begin{array}{rcl}{\rm{Y}} & = & 0.54+0.045{{\rm{X}}}_{{\rm{1}}}+0.075{{\rm{X}}}_{{\rm{2}}}+0.032{{\rm{X}}}_{{\rm{3}}}\\  &  & +\,0.073{{\rm{X}}}_{{\rm{1}}}{{\rm{X}}}_{{\rm{2}}}+0.058{{\rm{X}}}_{{\rm{1}}}{{\rm{X}}}_{{\rm{3}}}+0.098{{\rm{X}}}_{{\rm{2}}}{{\rm{X}}}_{{\rm{3}}}\\  &  & +\,0.033{{\rm{X}}}_{1}^{2}-0.077{{\rm{X}}}_{2}^{2}-0.10{{\rm{X}}}_{3}^{2}\end{array}$$

From Table 1, it is observed that under different extraction conditions, the overall flavonoid content in MME differed from 0.21 (Exp 9) to 0.67 mg/ml (Exp 8) (Table 1). These results indicated that the procedure conditions had relevance to the overall flavonoid content during the solvent extraction procedure since various processing conditions resulted in varied total flavonoid contents.

Among the three factors, extraction time (X_1_), solid-liquid ratio (X_2_) and extraction number (X_3_), only the linear coefficients of X_2_ were significant, which means that the solid-liquid ratio was the critical variable for the total flavonoid content from MME (*p* = 0.023) and **(**F-value = 8.42, the ratio of mean square ascribed to regression to the mean square to the real error); the interaction term of the solid liquid ratio to extraction times (X_1_X_3_) and the quadratic term of the extraction number (X_3_2) regression indicates that the model was significant, because the value of F-statistic (F-value = 4.50) and the low *P*-values (*p* = 0.032, *p* = 0.025).

The regression optimization response maps are shown in Fig. 1(A). The response curve shown in Fig. 1 shows that the factors and extraction of flavonoids have a significant quadratic parabolic relationship, indicating that the regression equation is good. The increase of the evaluation index increases with the size of the slope. Furthermore, the contour plot reflects the interaction between two factors, so that a weak interaction between two factors is indicated by a rounded contour, while a significant interaction is indicated by a distorted contour^22^.

According to the quadratic polynomial equation, the response curves and contour plots of the interaction items of each index and response value were drawn to determine the effect of each factor on total flavonoids. The order of influence of each factor on total flavonoids extraction was: extraction number < extraction time < solid-liquid ratio.

### Effects of the extraction parameters on the icariin content

The following equation can be used to describe the effects of the extraction number, solid-liquid ratio and extraction time on the icariin content:

$$\begin{array}{rcl}{\rm{Y}} & = & 8.800{\rm{E}}-003+5.875{\rm{E}}-004{{\rm{X}}}_{{\rm{1}}}-1.537{\rm{E}}-003{{\rm{X}}}_{{\rm{2}}}+5.250{\rm{E}}-003{{\rm{X}}}_{{\rm{3}}}\\  &  & +\,6.750{\rm{E}}-004{{\rm{X}}}_{{\rm{1}}}{{\rm{X}}}_{{\rm{2}}}+9.000{\rm{E}}-004{{\rm{X}}}_{{\rm{1}}}{{\rm{X}}}_{{\rm{3}}}-003-2.500{\rm{E}}\\  &  & -\,{{\rm{003X}}}_{{\rm{2}}}{{\rm{X}}}_{{\rm{3}}}+0{{\rm{.010X}}}_{1}^{2}-{\rm{3}}{\rm{.375E}}-{{\rm{004X}}}_{2}^{2}+3{\rm{.3138E}}-003{{\rm{X}}}_{{\rm{3}}}^{{\rm{2}}}\end{array}$$

Table 3 shows that model F-value of 13.49 and regression *p* value of 0.0003 indicates that the regression model is significantly reliable. A correlation coefficient of R^2^ = 0.9653 indicates that the equation is better and that the model can predict the MME extraction process.

In this study, the icariin content in MME differed from 0.0104 (Exp17) to 0.0265 mg/ml (Exp2) (Table 1).The independent variables of the extraction times showed that the extraction number had a significant impact on the extraction rate (F-value = 29.38, *p* = 0.001).

From Fig. 1(B), we know that by comparing the slope of the independent variables of the solid-liquid ratio, extraction time, and number of extraction procedures, the number of extraction procedures (X_3_) had a larger slope than the other two factors, showing a relatively steep curve. This indicates that the number of extraction procedures is the most significant factor that influences the extraction yield of the icariin content from MME.

The influence of the three factors on the extraction rate was solid-liquid ratio < extraction time < number of extraction procedures.

### Effects of the extraction parameters on the extraction yield

The following equation was used to describe the effects of the extraction number, solid-liquid ratio and extraction time on the extraction yield of MME:

$$\begin{array}{rcl}{\rm{Y}} & = & 28.73+1.31{{\rm{X}}}_{{\rm{1}}}+1.03{{\rm{X}}}_{{\rm{2}}}+4.20{{\rm{X}}}_{{\rm{3}}}\\  &  & +\,0.70{{\rm{X}}}_{{\rm{1}}}{{\rm{X}}}_{{\rm{2}}}+0.22{{\rm{X}}}_{{\rm{1}}}{{\rm{X}}}_{{\rm{3}}}+0.42{{\rm{X}}}_{{\rm{2}}}{{\rm{X}}}_{{\rm{3}}}\\  &  & -\,0.28{{\rm{X}}}_{1}^{2}-0.26{{\rm{X}}}_{2}^{2}-2.00{{\rm{X}}}_{3}^{2}\end{array}$$

Table 3 shows the model F-value of 36.95 and model regression *p* value of 0.0001, indicating that the regression model was significantly reliable; the correlation coefficient of R^2^ = 0.9794 indicates that the equation was better and that the model can predict the MME extraction process.

These three independent variables (solid-liquid ratio, extraction times, number of extractions) had a highly significant impact on the extraction rate (F-value = 24.93, F-value = 15.28, F-value = 254.46; *p* = 0.002, *p* = 0.006, *p* = 0.001).

From the response curve shown in Fig. 1(C), it is observed that the number of extractions (X_3_) had the most significant effect on the extraction rate, with a relatively steep curve, followed by extraction time (X_1_) The solid-liquid ratio (X_2_) had a smoother curve with a smaller response value as its value increased or decreased. The influence of the three factors on the extraction rate was solid-liquid ratio < extraction time < number of extractions. The square of the number of extractions had a significant impact on the experimental results **(**F-value = 30.49, *p* = 0.001).

### PDE 5 inhibition rate of MME

The following equation was used to describe the effects of the number of extractions, solid-liquid ratio and extraction time on the PDE 5 inhibition rate (Y_3_) of MME:

$$\begin{array}{c}{\rm{Y}}=36.46+0.59{{\rm{X}}}_{{\rm{1}}}-0.29{{\rm{X}}}_{{\rm{2}}}+6.13{{\rm{X}}}_{{\rm{3}}}+0.80{{\rm{X}}}_{{\rm{1}}}{{\rm{X}}}_{{\rm{2}}}-6.88{{\rm{X}}}_{{\rm{1}}}{{\rm{X}}}_{{\rm{3}}}\\ \,\,\,+\,3.68{{\rm{X}}}_{{\rm{2}}}{{\rm{X}}}_{{\rm{3}}}+18.37{{\rm{X}}}_{1}^{2}+17.42{{\rm{X}}}_{2}^{2}+13.80{{\rm{X}}}_{3}^{2}\end{array}$$

In our work, under diverse extraction conditions, the PDE5 inhibition rate in MME varied from 36.2% to 82.1%. Table 3 shows that the model F-value of 9.70 and model regression *P* value was 0.0034, indicating that the regression model is significant and reliable; the correlation coefficient of R^2^ = 0.9257 indicates that the equation is appropriate and the model can predict the MME extraction process. For the three independent variables, the number of extraction times had a significant effect on the inhibition rate of PDE5 **(**F-value = 15.70, *p* = 0.004**)**.

From the response curve shown in Fig. 1(D), the slope of the response surface showed an upward tendency with an increase in the number of extractions and the contour plot was distorted. We observe that the number of extractions (X_3_) for PDE5 is the most significant, showing a relatively steep curve; while the extraction time (X_1_) and solid-liquid ratio (X_2_) did not have any significant effect on the inhibition rate of PDE5, and the step curves were smoother and the contour plots were rounded.

Furthermore, all four responses were useful in the development of the desirability function. By using the desirability function approach, the optimized conditions within the studied variables’ experimental range were the number of extractions, 1.19, extraction time, 2 h, and solid-liquid ratio, 1:16. At this point, the predicted values and experimental values of the system were computed as total flavonoids of 0.47 mg/ml to 0.49 mg/ml, icariin content of 0.026 mg/ml to 0.028 mg/ml, extraction yield of 26.00% to 26.50% and PDE5 inhibition rate of 81.07% to 84.10%.

### Weight change

**Figure 3:**
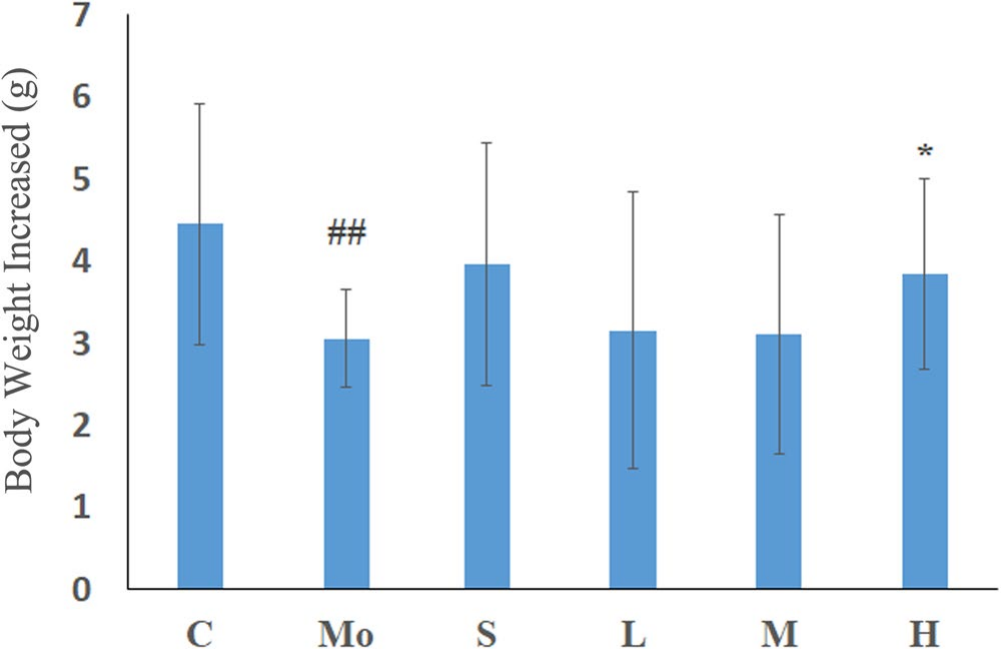
Effects of MME on the body weight increase. C: control group; Mo: model group; S: sildenafil treatment group; H: high dose treatment group; M: middle dose treatment group; L: low dose treatment group. Data represent the mean ± S.D. n = 7–10. ^##^*p* < 0.01 vs. control group, *p < 0.05 vs. model group.

See Fig. 3; after modelling with hydrocortisone, there was a decrease in activity, unresponsiveness, and sparse hair loss in the model animals. Their weight gain slowed, and their body weight gain was significantly lower than that of the control group at 21 days. In each treatment group, the above symptoms were relieved and the weight gain was higher than that of the model group. There was a significant difference between the high dose group and model group (*p* = 0.049), indicating that MME can improve the “depletion”phenomenon caused by hydrocortisone.

### Mating Ability

**Figure 4:**
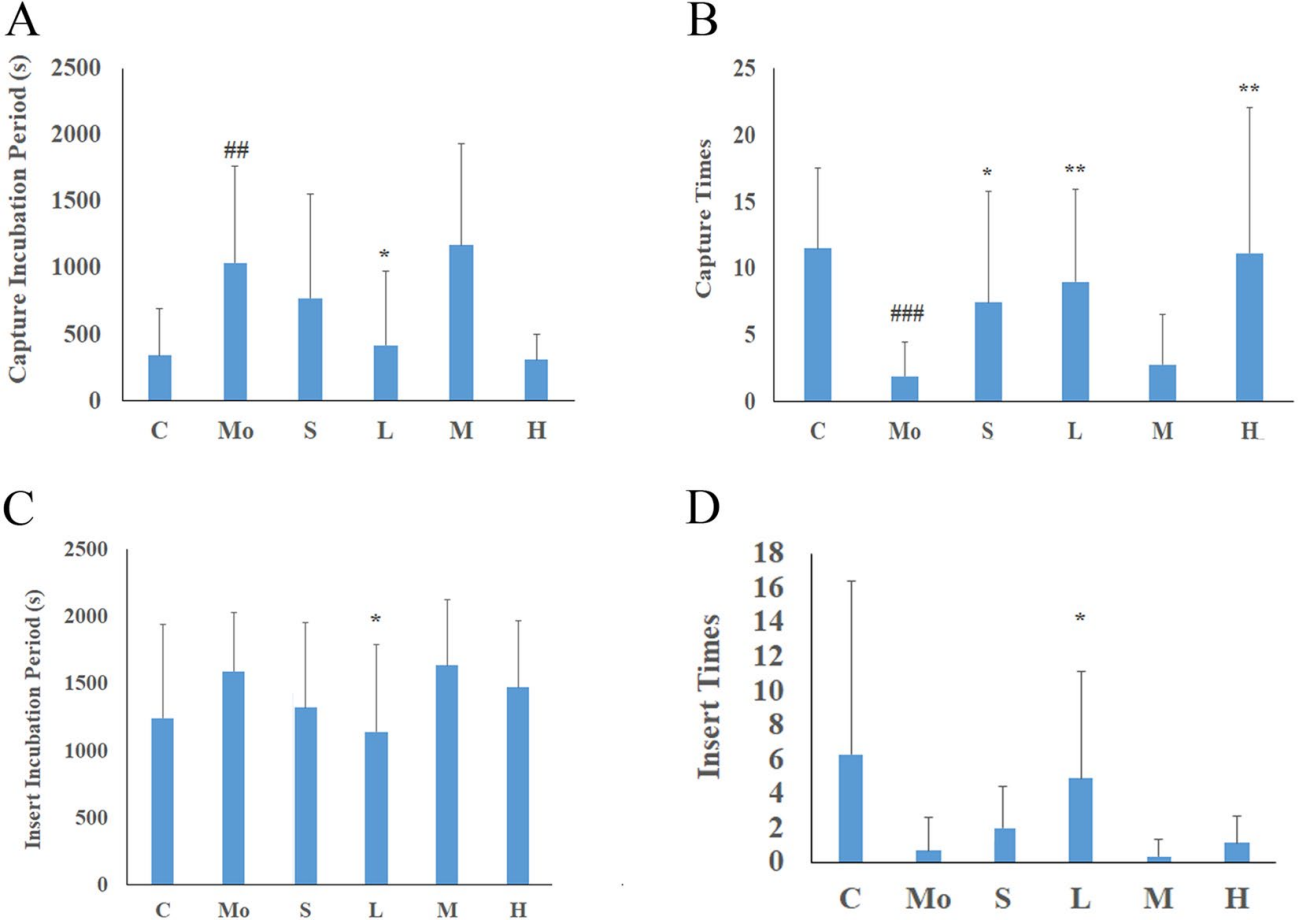
Effects of MME on the capturing and inserting ability of mice treated with hydrocortisone (n = 7–10). (**A**) Capture incubation period. (**B**) Capture times within 30 minutes. (**C**) Insert incubation period. (**D**) Insert times within 30 minutes. C: control group; Mo: model group; S: sildenafil treatment group; H: high dose treatment group; M: middle dose treatment group; L: low dose treatment group. Data represent the mean ± S.D. n = 7–10. ^##^*p* < 0.01, ^###^*p* < 0.001 vs. control group, *p < 0.05, **p < 0.01 vs. model group.

From the above results (Fig. 4), it is observed that the mouse capture incubation period was prolonged by 204.64% (*p* = 0.007) after modelling and that the number of capture times within 30 minutes decreased by 83.48% (*p* = 0.0001); there was a decrease in the percentage of participating captured animals by 40% and an insert incubation period extension of 28.03%. The number of insertion times over 30 minutes decreased by 88.89%, but there was no significant difference; the percentage of animals involved in insertion decreased by 66.67%.

After administration, the capture incubation period of the lower-dose group was reduced by 59.71% (*p* = 0.028) and those of the sildenafil group, low and high dose group demonstrated an increase in the number of captures within 30 minutes (291.81%, 373.68%, 486.47%, respectively; *p* = 0.031, *p* = 0.028, *p* = 0.049**)** where the high and low dose groups were *p* = 0.0099, *p* = 0.004) in comparison with the model group. The high dose group percentage increased for the number of capture times over 30 minutes by 66.67%, the low dosage group had an insertion latency reduction of 28.24% (*p* = 0.046), and the low-dose group had increased insertion times over 30 minutes by 598.41% (*p* = 0.021), with an increased percentage of participants involved in inserting animals over 30 minutes of 233.35% (*p* = 0.030).

The results show that sildenafil, low, medium and high doses of MME can improve the mating ability of mice after hydrocortisone treatment, shorten the time of sexual arousal, and increase the number of sexual activities, indicating that they can counteract the adverse effects of hydrocortisone on the mating ability of mice.

### Organ index and testicular tissue morphology

**Figure 5:**
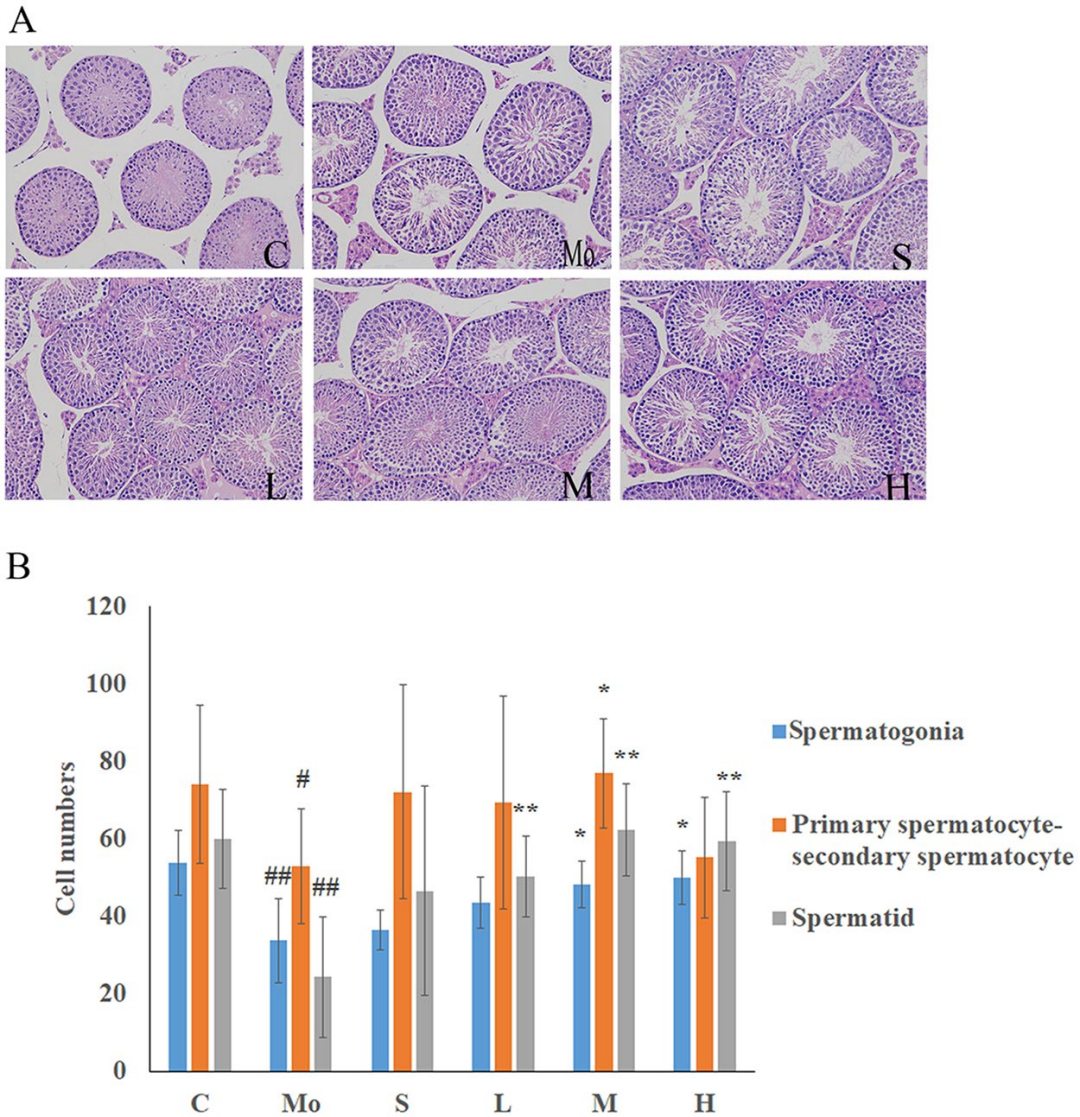
Effects of MME on the testicular tissue of mice treated with hydrocortisone. (**A**) HE staining results, 200×. (**B**) Germ cells count. C: Control group; Mo: model group; S: sildenafil treatment group; H: high dose treatment group; M: middle dose treatment group; L: low dose treatment group. Data represent the mean ± S.D. n = 5. ^#^*p* < 0.05, ^##^*p* < 0.01 vs. control group; *p < 0.05, **p < 0.01 vs. model group.

The results are shown in Table 5 and Fig. 5.

**Table 5:** MME effect on the organ index of kidney-yang deficiency mice (MEAN ± SD).

Group (n = 10)	Organ index (mg weight)					
	Adrenal	Semen	Testis	Epididymis	Preputial gland	Penis
Control group	0.17 ± 0.07	5.99 ± 1.57	6.82 ± 1.09	2.79 ± 0.39	3.26 ± 0.77	1.38 ± 0.14
Model group	0.15 ± 0.06	4.27 ± 1.48^#^	7.62 ± 1.05	2.57 ± 0.40	2.99 ± 0.58	1.28 ± 0.23
Sildenafil	0.18 ± 0.08	5.66 ± 2.13	7.24 ± 1.54	2.49 ± 0.52	2.93 ± 0.83	1.37 ± 0.34
Low-dose group	0.20 ± 0.12	5.40 ± 1.44	7.27 ± 1.63	2.70 ± 0.40	3.68 ± 0.96*	1.40 ± 0.30
Middle dose group	0.23 ± 0.10*	5.02 ± 1.31	8.14 ± 1.35	2.88 ± 0.45	3.13 ± 0.49	1.35 ± 0.20
High-dose group	0.25 ± 0.07**	5.69 ± 1.50*	7.74 ± 0.83	2.69 ± 0.43	3.36 ± 0.45	1.58 ± 0.12**

In comparison with the control group, ^#^*p* < 0. 05; in comparison with the model groups, **p < 0.01.

After modelling, the weight index of semen was significantly reduced (*p* = 0.017) and the weight indexes of the adrenal, epididymis, preputial gland and penis decreased, but without significant differences. The MME low dose group of the model group mice adrenal weight index increased, and the middle and high dose group had a significantly increased model group mice adrenal weight index (*p* = 0.047, high-dose group, *p* = 0.008).

The MME low and middle dose groups demonstrated positive effects on the weight index of semen in mice with a model group, but without a significant difference. The high dose group demonstrated a significant increase in the weight index of semen in mice with a model group (*p* = 0.045). In the low dose group, the weight index of the preputial gland in model group was significantly increased (*p* = 0.049). The high dose group had a significantly increased penile weight index in model group (*p* = 0.010) (Table 5). These results show that MME has a positive effect on the reproductive organs (*p* < 0.05, *p* < 0.01) and on the damage caused by hydrocortisone to the seminal vesicle to some extent.

Figure 5 shows testicular paraffin embedded sections of the HE staining results, in which the control group mice testicular seminiferous tubules were regular and round and arranged in neat rows and the spermatogonium were large and round, close to the seminiferous tubule basement membrane, followed by the inner side of the primary spermatocytes, secondary spermatocytes and sperm cells. In the testes of the model group, the seminiferous tubules had increased voids in the lumen and a sparse structure; in the MME low, medium and high dose groups, this phenomenon showed some improvement.

### Serum testosterone levels

**Figure 6:**
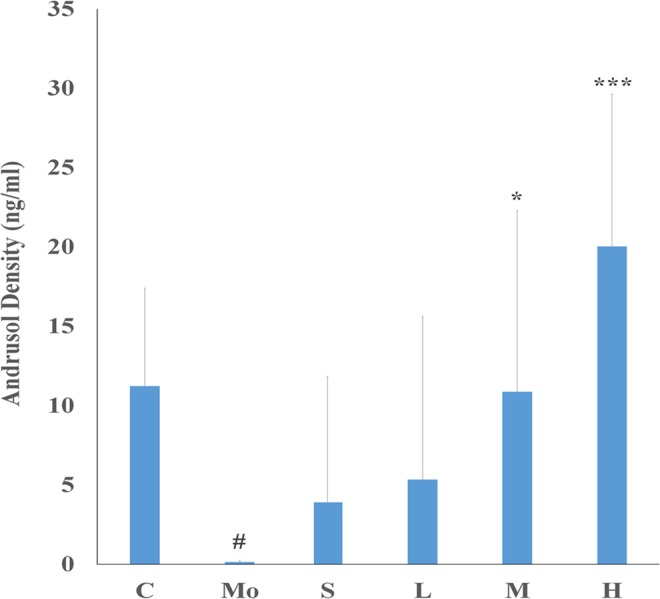
Effects of MME on serum testosterone of mice treated with hydrocortisone. C: control group; Mo: model group; S: sildenafil treatment group; H: high dose treatment group; M: middle dose treatment group; L: low dose treatment group. Data represent the mean ± S.D. n = 5. ^#^*p* < 0.05, ^##^*p* < 0.01 ^###^*p* < 0.001 vs. control group; *p < 0.05, **p < 0.01, ***p < 0.001 vs. model group.

Compared with the control group (Fig. 6), the testosterone level in the model group was reduced by 98.75% (*p* = 0.022) after the model was established. The levels of serum testosterone in the middle-dose group and high-dose group, were increased by 7664.29% (*p* = 0.015) and 14207.14% (*p* = 0.0001), respectively, compared to the model (Fig. 6), which demonstrates that MME has a significant positive effect on the hypothalamus- pituitary- gonadal axis.

### Penile tissue PDE5 expression levels

**Figure 7:**
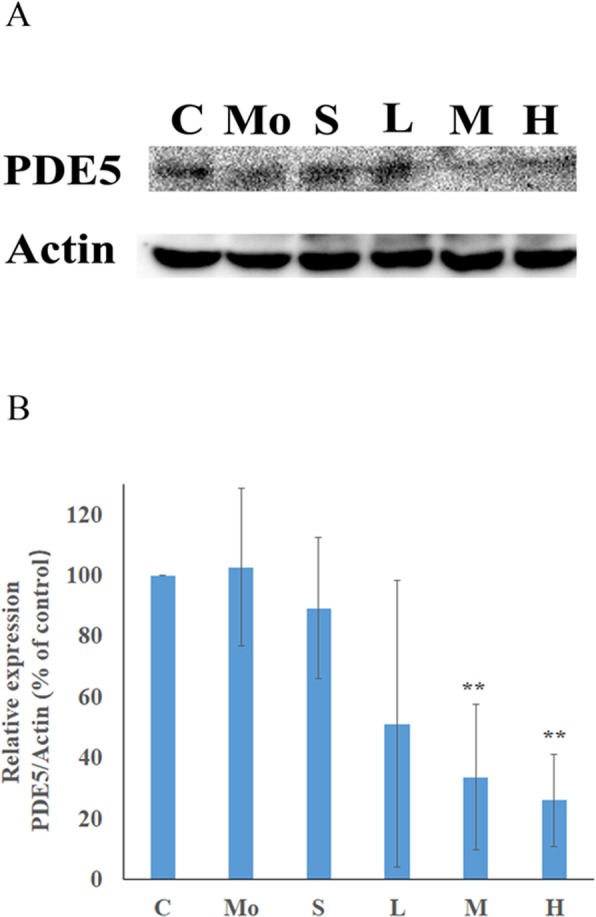
Effects of MME on the PDE5 expression level in the penis of mice treated with hydrocortisone. (**A**) Western blotting was used to detect the PDE5 protein expression levels. (**B**) Densitometry analysis is presented as the relative ratio of protein PDE5/actin using Quantity One software. C: control group; Mo: model group; S: sildenafil treatment group; H: high dose treatment group; M: middle dose treatment group; L: low dose treatment group. Data represent the mean ± S.D. n = 3. ^**^p < 0.01, vs. model group.

From Fig. 7 we can see that after modelling, no significant alteration was found in the expression of PDE5 in the penile tissue of the model group compared with the control group. However, after administration, the PDE5 expression level in the penile tissue of the middle-dose group and high-dose group were lower than that of the model group by 67.22% (*p* = 0.004) and 74.69% (*p* = 0.001). This demonstrates that MME can significantly reduce the expression of PDE5 in penile tissue.

In order to further explore the aphrodisiac effect of MME *in vivo*, kidney Yang deficiency syndrome mice model were established with hydrocortisone. After modelling with hydrocortisone, compared with the control group, the weight loss of mice was significantly reduced, which is the typical “depletion” phenomenon. In terms of mating ability, hydrocortisone can prolong sexual arousal in mice and reduce the number of sexual encounters. In addition, hydrocortisone modelling also led to varying degrees of decreases of reproductive organs’ indexes, as well as pathological damage to the testicular tissue structure. The above results showed that a subcutaneous hydrocortisone injection in mice successfully led to kidney yang deficiency^23^. After administration of MME, the body weight gain of mice increased slightly, relieving the “depletion”caused by hydrocortisone. At the same time, after the administration of MME, the time of sexual awakening was reduced and the number of sexual encounters increased. These results demonstrate that MME significantly improved the mating ability of mice with impotence due to kidney yang deficiency. In addition, MME reduced hydrocortisone-induced contraction of various organs of the reproductive system and fought the pathological damage caused by the testicular tissue structure as well as increased seminiferous tubules spermatogonia, primary spermatocytes, secondary sperm, and number of cells and sperm cells. The above results show that from different aspects, MME, in a hydrocortisone-induced mouse model of kidney yang deficiency, has an aphrodisiac effect.

Modern studies have shown that the pathological changes caused by kidney yang lead to deficiency dysfunction of the hypothalamus-pituitary-gonadal (HPG) axis. The hydrocortisone-induced animal model of kidney yang has reduced serum testosterone levels^24^. The experimental results showed that the mice serum testosterone levels of the model group were significantly reduced and after administration of MME the serum levels of testosterone were increased, especially in the difference was found in the middle and high dose groups. In addition, MME in mice increased the serum testosterone levels in a dose-dependent manner. The above results showed that MME can promote the secretion and release of testosterone, inducing an aphrodisiac effect. The mechanism of sildenafil inhibition of PDE5 activity is unrelated to the serum testosterone levels, and in our results, sildenafil treatment of mice had no significant effect on serum testosterone.

The *in vitro* experimental results showed that MME has PDE5 inhibition activity. On this basis, we examined the effect of MME on penile PDE5 expression in mouse models of hydrocortisone-induced kidney yang deficiency. The results showed that hydrocortisone treatment did not affect the mouse penile PDE5 expression levels and that MME significantly inhibited the expression of PDE5. At the same time, the PDE5 activity inhibitor sildenafil, although it can inhibit PDE5 activity, did not significantly inhibit the level of expression of PDE5. The above results show that MME not only has inhibitory activity towards PDE5 but also significantly inhibits the expression of PDE5 in the penis. Although in this study, we have obtained pleasant results, but using drugs to create animal models similar to kidney-yang deficiency will still have some disparities with the actual kidney-yang deficiency, the link between traditional and modern diagnosis of ED cannot be guaranteed, also, translation from animal results to human are not straightforward.

## Conclusion

RSM was successfully applied for extraction of total flavonoids and analysed according to the icariin content, extraction yield and PDE5 inhibition activity of MME. A mono factor experiment was used to fit the number of extractions, extraction time and solid to liquid ratio. The regression model of the quadratic polynomial mathematical model of the yield was tested on these three factors of MME extraction. The model is reasonable and reliable and can accurately predict the extraction process of MME combined with its actual production. It was determined that the optimal extraction process was a solid-liquid ratio of 1:16, extraction time of 2 h, extraction number of 1. The system’s predicted responses were computed to be total flavonoids, 0.49 mg/ml; icariin content, 0.028 mg/ml; targeted extraction yield, 26.50%; and PDE5 inhibition rate, 84.10%. Fitted towards a second-order quadratic polynomial model, the experimental consequences a good suitability to the model proposed (R^2^ > 0.90 and R^2^ > 0.85). This study can be used in the extraction of MME and can provide scientific data for its reasonable development as a natural PDE5 inhibitor agent.

In hydrocortisone-induced mouse yang deficiency model, MME induced a significant improvement in sexual function, potential to become ED therapies for the clinical management of patients with kidney yang deficiency and its mechanism may be related to increasing serum testosterone, inhibiting PDE5 activity and inhibiting penile PDE5 expression.

## Materials and Methods

### Plant material and chemicals

*Epimedium brevicornu* Maxim (batch number: 201609003), *Ana-cyclus pyrethrum* (L.) DC (batch number: 201608003), *Lycium barbarum* L (batch number: 201607003), *Cuscuta auctralis* R.Br (batch number: 201607003) and other plants were purchased from the Hospital of Traditional Uyghur Medicine in Xinjiang. Anwar Talip, the director of the pharmaceutical department, identified these as authentic medicine. Icariin (batch number: 110737-201516, purity: >98%) was acquired from the Chinese Food and Drug Accreditation Institute, as were methanol, acetonitrile chromatographic grade (US Tedia), and high-purity water.

### Determination of total flavonoid content

Ultraviolet-visible spectroscopy^25^ was used to measure the overall flavonoid content. The preparation process of the test sample solution was as previously reported^26^. Rutin was utilized as the standard for the calibration curve. Total flavonoid estimation was conducted in triplicate. The consequences were presented as rutin equivalents per millilitre of liquid extracts (mg of RE/ml). The flavonoid content of the mixture was compared to the rutin standard curve (Y = 0.0118X + 0.0002, R^2^ = 0.9999, 0.018 − 0.107 mg/ml).

### Determination of icariin content

High-performance liquid chromatography (HPLC-UV) (Agilent Technologies, 1200, Alto, CA, USA) was used to measure the icariin content^27^. The samples were then chromatographed under the following chromatographic conditions: chromatographic column (150 × 4.6 mm, 5 μm) C_18_ (Therma); Mobile phase A, acetonitrile; mobile phase B, phosphoric acid in water (0.1%) and (A:B = 29:71, v/v); flow rate, 1.0 ml/min; injection volume, 10 μl; stop time, 60 min; and column temperature, 30 °C, with UV detection at 270 nm.

### Determination of Extraction Yield

Fifty millilitres of the test solution was placed in a constant weighted crucible pot, evaporated in a water bath, dried at 105 °C for more than 3 h, moved to the dryer, cooled for 30 min, and quickly weighed^28^.

### Determination of PDE5 inhibitory activity *in vitro*

The advanced scintillation proximity assay technique (SPA)^29^ was used to carry out the study. Samples were prepared with 10 mg/L or 10 mM stock solution in dimethyl sulphoxide (DMSO) and then diluted with distilled water to the concentration to be measured. The positive control sildenafil citrate tablets were prepared with a 10^−2^ M concentration of stock solution and then diluted with distilled water to a concentration of 10^−5^ M. The details of experimental steps are shown in Table 6.

**Table 6:** PDE5 inhibition activity assay procedure. (Control: sildenafil citrate tablets; sample: MME). Mix well and allow to stand at room temperature for 20 minutes, Count in SPC 1450 Micro Beta Tri lux.

	Control	Sample	Blank
10x assay buffer	10 *μ*l	10 *μ*l	10 *μ*l
PDE enzyme	10 *μ*l	10 *μ*l	—
Ultrapure water	60 *μ*l	60 *μ*l	70 *μ*l
Inhibitor sample	—	10 *μ*l	—
Inhibitor diluents	10 *μ*l	—	10 *μ*l
[3 H] cGMP	10 *μ*l	10 *μ*l	10 *μ*l
**Incubate for 20 minutes at 30 °C**			
SPA beads	50 *μ*l	50 *μ*l	50 *μ*l

### Mono-factor experiments

The solid-liquid ratio, extraction number, and extraction time are the main factors that might have an influence on extraction efficiency. The impacts on the total flavonoid content, icariin content, extraction yield, and PDE5 inhibition activity of MME caused by these individual factors were assessed by mono-factor experiments. The following shows the parameters along with their ranges: extraction time (1, 1.5, 2 h), solid-liquid ratio (1:12, 1:14, 1:16), and extraction number (1, 2, 3 times). The three extraction methods and three levels of BBD response surface extraction technology were selected. The levels and factors are shown in Table 7.

**Table 7:** Uncoded and coded levels of the independent variables used in the RSM design.

Symbol	Independent variable		Coded levels	
X_1_	Extraction time	−1 1	0 1.5	1 2
X_2_	Solid: liquid ratio	12	14	16
X_3_	Extraction number	1	2	3

X_1_ = Extraction time (h), X_2_ = Solid-liquid ratio (multiple), X_3_ = Extraction number.

### Response surface methodology (RSM)

Recently, various areas of science have used RSM, and its efficiency and feasibility have been highlighted by some study groups. In the current study, RSM was utilized to optimize the extraction procedure coupled with BBD^30,31^, which is an experimental design that fits the second order response surface on the basis of the construction of a balanced imperfect block design^32,33^.

### Animals and grouping

All male ICR mice weighing 18–22 g were purchased from the Department of Laboratory Animal Science of Peking University Health Science Center (Beijing, China). All mice were housed under standardized housing conditions (12/12 h light/dark cycle; temperature, 22 ± 2 °C; relative humidity, 50 ± 5%) and were provided food and water *ad libitum*. All animal-related procedures were approved by the Animal Care and Use committee of Peking University Health Science Center and the methods were carried out in accordance with the relevant guidelines and regulations.

Experimental mice were randomly divided into 6 groups: Control group (n = 10, equal volume of normal saline), Model group (n = 10, equal volume of normal saline), sildenafil group (n = 10, sildenafil 10 mg/kg), and MME low (n = 10, MME 0.13 g/kg), medium (n = 10, MME 0.26 g/kg) and high dose groups (n = 10, MME 0.52 g/kg). Mice from each group were daily given corresponding drug by gavage for 21 consecutive days.

### Kidney yang deficiency modelling method

According to the method reported in the literature, subcutaneous injection of hydrocortisone was used to produce a kidney yang deficiency model^23^. From the first day to the seventh day 1 hour before MME administration, animals in the model group, sildenafil group, and low, medium and high dose groups were treated with 25 mg/kg subcutaneous injections of hydrocortisone for 7 days; the control group was injected with an equal volume of normal saline.

### Promoting oestrus in female mice

Adult female mice 72 and 48 hours before the mating experiment were subcutaneous injected with 0.144 mg/kg estradiol benzoate, and 5 hours before mating, they were injected with a 0.722 mg/kg dose of medroxyprogesterone ketone to synchronize their oestrus time with the mating experiment^34^.

### Mating ability indicator observations

On the 21st day of administration, 1 h after the last administration to each group of animals, male rats were placed in a squirrel cage for 10 minutes and then placed with oestrus females. The mice were monitored by an infrared camera, observed and recorded. The time since first injection of the females was the incubation period, insertion time since first insertion was the incubation period, and number of insertions that occurred within 30 minutes was recorded^35^.

### Organ index and testicular tissue observation

The animals were killed 24 hours after the last administration, and the adrenal gland, seminal vesicle, testis, epididymis, and preputial gland and penis were weighed, and the organ index (mg organ weight/g body weight) was calculated. One side of the testis was fixed with 10% paraformaldehyde, embedded in paraffin, sliced and stained with HE. On regular images magnified 200 times, 25 regular round microtubules were selected, and their diameters were measured with Image Pro Plus software. Choosing from five complete rounds of seminiferous tubules, we counted the spermatogonia, primary spermatocytes, secondary spermatocytes, and spermatids^36^.

### Serum testosterone content determination

Within 24 hours of the last administration, orbital-plexus blood was collected and centrifuged for 15 mins at 3000 rpm and then left to stand at 4 °C for 1 h. Serum was separated and stored at −80 °C. Enzyme-linked immunosorbent assay (ELISA) was used to determine the testosterone concentrations.

### Penile tissue PDE5 expression levels

Total protein was extracted from the penile tissue and quantified by the BCA method. Each lane was loaded with 30 μg of protein and separated by SDS-PAGE using an 8% dissociation gel. Afterwards, the cells were transferred to PVDF membranes, blocked with 5% non-fat milk for 1 h at 37 °C, and incubated with the primary antibody anti-PDE5A (ab14672, Abcam, USA 1: 200 dilution) and anti-actin (sc-1616-R, Santa Cruz, USA 1:200 dilution). There were then incubated with the corresponding secondary antibodies (IS003, MACGENE, CHN, 1:2000 dilution). Luminescence using ECL substrate was measured using exposure photography with a Bio-Rad imaging system, followed by grading of bands by Quantity one, and the PDE5 levels were measured using actin as an internal reference^37–39^.

### Statistical analysis

T tests and design-Expert v.8.06 trial (Stat-E-ase, Minneapolis, Minnesota, USA) were used to perform statistical analyses. The use of an empirical model was used to try to better understand the correlations between the response and factors by employing a second-order polynomial quadratic model as Eq. 1 shows,

$$Y={B}_{0}+\sum _{i=1}^{n}{B}_{i}{X}_{i}+\sum _{i=j=1}^{n}{B}_{ij}{X}_{i}{X}_{j}$$

in which Y stands for response variables (total flavonoid, icariin content, PDE5 inhibitory activity, extraction yield); X_i_ and X_j_ represents independent variables (extraction numbers, solid-liquid ratio, extraction time); β I j, βii, βI and β0 and represent the regression coefficients of interaction, quadratic, linear, and intercept coefficient, respectively.

where n = 3, the equation (1) can be converted to:

$${\rm{Y}}={{\rm{B}}}_{{\rm{0}}}+{{\rm{B}}}_{{\rm{1}}}{{\rm{X}}}_{{\rm{1}}}+{{\rm{B}}}_{{\rm{2}}}{{\rm{X}}}_{{\rm{2}}}+{{\rm{B}}}_{{\rm{3}}}{{\rm{X}}}_{{\rm{3}}}+{{\rm{B}}}_{{\rm{12}}}{{\rm{X}}}_{{\rm{1}}}{{\rm{X}}}_{{\rm{2}}}+{{\rm{B}}}_{{\rm{13}}}{{\rm{X}}}_{{\rm{1}}}{{\rm{X}}}_{{\rm{3}}}+{{\rm{B}}}_{{\rm{23}}}{{\rm{X}}}_{{\rm{2}}}{{\rm{X}}}_{{\rm{3}}}+{{\rm{B}}}_{{\rm{11}}}{{\rm{X}}}_{1}^{2}+{{\rm{B}}}_{{\rm{22}}}{{\rm{X}}}_{2}^{2}+{{\rm{B}}}_{{\rm{33}}}{{\rm{X}}}_{3}^{2}$$

in which *B*_0_ represents a constant term; *B*_1_, *B*_2_, and *B*_3_ are linear coefficients; *B*_12_, *B*_13_, and *B*_23_ are interaction term coefficients; and *B*_11_, *B*_22_ and *B*_33_ are quadratic coefficients.

### Animal use Approval Statement

All animal experiments were conducted according to the principles of the National Institutes of Health (NIH) Guide for the Care and Use of Laboratory Animals and were approved by the ethics committee for laboratory animal care and use of Peking University Health Science Center. All efforts were made to minimize the suffering of the animals used in this study.

## Supplementary information

Supplementary information-SREP-18-07207A-Ayinuer Reheman.pdf

## Data Availability

All data generated and analyzed during this study are included in this published article and it’s Supplementary Information.
