# Supplementary material for: Optimization of Extraction Technology of Majun Mupakhi Ela and its Effect on Hydrocortisone-induced Kidney Yang Deficiency in Mice
Source: Sci Rep. 2019 Mar 15;9:4628. doi: 10.1038/s41598-019-41006-6 (PMC6420599; doi:10.1038/s41598-019-41006-6)
Supplement: Supplementary file 1 — Supplementary information-SREP-18-07207A-Ayinuer Reheman.pdf [file 41598_2019_41006_MOESM1_ESM.pdf]

## Supplementary Information

## Manuscript number: SREP-18-07207A

# Optimization of Extraction Technology of Majun Mupakhi Ela and its Effect on Hydrocortisone-induced Kidney Yang Deficiency in Mice

Ayinuer Reheman[1, 2, 4, #], Ze-yu Gao[3, #], Xirali Tursun[4], Xiao-Ping Pu[3], Tao WU[1], Fei He[1], Xin Zhao[3,*], and Haji Akber Aisa[1,*]

[#] These two authors contributed equally to this work

[*] Co-corresponding authors

[1] Key Laboratory of Plant Resources and Chemistry in Arid Regions, Xinjiang Technical Institute of Physics and Chemistry, Chinese Academy of Sciences, 830011, Urumqi, Xinjiang, China. [2] College of Traditional Uyghur Medicine, Xinjiang Medical University, 830011, Urumqi Xinjiang, China. [3] Department of Molecular and Cellular Pharmacology, School of Pharmaceutical Sciences, Peking University, Xue yuan Road 38, Beijing, P. R. China. [4] University of Chinese Academy of Sciences, 100039. Beijing, China. Correspondence and requests for materials should be addressed to H.A. Aisa (email: haji@ms.xjb.ac.cn)and X. Z. (zhaoxin2010@bjmu.edu).

**Supplementary Table A1 Effect of MME on Testis Tissue in Kidney-Yang Deficiency Mice（Mean±SD，n=5）**

| Group | Trichostatin tube diameter(mm) | Spermatogonial cells ( number ) | Primary spermatocytes - Secondary spermatocytes （number） | Sperm cells （number） |
|---|---|---|---|---|
| Control group | 0.20±0.02 | 54.00±8.37 | 74.20±20.49 | 60.00±12.71 |
| Model group | 0.20±0.01 | 33.80±10.92## | 53.00±14.93# | 24.40±15.61## |
| Sildenafil | 0.19±0.01 | 36.40±5.13 | 72.20±27.51 | 46.60±27.01 |
| Low-dose group | 0.20±0.01 | 43.60±6.73 | 69.40±27.56 | 50.40±10.41** |
| Middle dose group | 0.20±0.01 | 48.40±6.02* | 77.00±14.09* | 62.40±12.01** |
| High-dose group | 0.19±0.01 | 50.00±6.89* | 55.20±15.45 | 59.40±12.82** |

Compared with the control group，# $p<0.05$，## $p<0.01$；Compared with the model group，* $p<0.05$，** $p<0.01$。

As shown in supplementary table A1, after modeling, the number of spermatogenic cells at all levels significantly decreased ($p$ <0.05), the number of spermatogonia and the number of sperm cells ($p$ <0.01), after administration, the number of spermatogenic cells in each group increased ($p$ <0.01) and the number of spermatogenic cells in the middle dose group was significantly different from that in the model group ($p$ <0.05), in which sperm ($p$ <0.01). The number of spermatogonia and the number of sperm cells in high dose group were significantly different from those in model group ($p$ <0.05, $p$ <0.01, respectively).

The above results show that, MME to some extent, against hydrocortisone pathological effects on testicular tissue
